# White epidermoid: A diagnostic dilemma

**DOI:** 10.1016/j.radcr.2025.03.081

**Published:** 2025-04-24

**Authors:** Poornima Maravi, Vijay Kumar Verma, Rambharat Bairwa, Anita Uikey

**Affiliations:** Department of Radiology, Gandhi Medical College and Hamidia Hospital, Bhopal, Madhya Pradesh, India

**Keywords:** MRI, Cyst, Epidermal, Dermoid cyst

## Abstract

A 27-year-old female patient presents with a chronic headache. Physical examination and laboratory tests show no remarkable abnormality. MRI brain with contrast was ordered for further evaluation of symptoms. MRI revealed a large extra-axial, posterior fossa base T1 hyperintense and T2 hypointense lesion. The Lesion showed FLAIR hypointensity with no significant diffusion restriction on DWI. Post contrast scans show no contrast enhancement. Based on the T1 hyperintensity, lesions with hyperintense contents were kept in differential diagnosis such as dermoid and proteinaceous cyst. However, the lesion demonstrated T2 and flair hypointensity suggesting a highly viscous contents within the lesion. The loss of diffusion restriction ruled out any possibility of classical epidermoid cyst.

The patients was kept on follow up with suggestion to remove the lesion surgically, although patient denied for surgical management and kept on symptomatic treatments with painkillers and multivitamins. This case report highlights the diagnostic dilemma in forming an MRI based diagnosis with dictation of a phenomenon where a lesion can exhibit a opposite character rather than exhibiting a classical intensity based on its contents. We can encounter a completely different imaging appearance of a lesion than what we thought to and should be kept in mind. This case report also highlights the fact that although the histopathology is main study of diagnosis and treatment in many cases it cannot be achieved in every case and management could rely purely on imaging findings.

## Introduction

Epidermoid cyst are non-neoplastic, true, inclusion cysts derived from ectoderm surrounded by stratified squamous epithelium and can be seen involving any part of the body including intracranial epidermoid cysts. The pseudocysts are lined by connective or granulation tissue instead of squamous epithelium. These inclusion cysts may present as a slowly growing, palpable mass with or without pain. In small number of cases they can transform to a malignancy in with incidence of 0.011%-0.045% [1,2]. They account for approximately 85%-95% of all excised cysts and can release macerated keratin .The term sebaceous cyst is now obsolete as these cysts do not contain sebaceous glands and but made up of proliferating surface epidermoid cells within the dermis. The other synonyms are infundibular cysts, epidermal cysts, epidermal inclusion cysts and epidermoid inclusion cysts [3]. The term dermoid cyst is also used extensively in this context however dermoid cysts have skin appendages on their walls while epidermoid cysts lack them. The clinical behavior of both cysts also differ as dermoid cysts are common and present during infancy or early childhood, usually located superficially and can mold the underlying bone with its destruction. In contrast to that, epidermoid cysts can be located anywhere and are diagnosed later in life [4].

These lesions are diagnosed when they become symptomatic and definitive diagnosis is made after excision .The extent of excision depends upon the adherence of tumor capsule to the surrounding vital structures [5]. The microscopic appearance shows a cystic lesion lined by cornified epithelium containing lamellated keratin without calcifications. They can be seen as hyperintense lesions on T2/FLAIR sequence of MRI.

### Case report

A 27-year-old female patient presented to the outpatient department with a history of persistent, severe headaches over the past 3 months. The headaches were predominantly localized to the bilateral occipital regions and were associated with intermittent nausea but no vomiting, visual disturbances, or focal neurological deficits. There was no history of fever, trauma, seizures, or altered sensorium. The patient had no prior history of major illnesses, systemic diseases, or neurological disorders. She had no known allergies and had not undergone any prior surgical procedures. There was no history of prior hospital admissions. The family history was unremarkable, with no known cases of intracranial tumors, genetic disorders, or malignancies among immediate relatives. There was no history of neurocutaneous syndromes such as Gorlin syndrome or Gardner syndrome, which are associated with epidermoid cysts.

On physical examination, the patient was hemodynamically stable with a blood pressure of 118/76 mmHg, heart rate of 72 beats per minute, and respiratory rate of 16 breaths per minute. Neurological examination revealed no cranial nerve deficits, motor weakness, or signs of increased intracranial pressure. Fundoscopic examination did not show papilledema.

Routine blood tests, including complete blood count (CBC), liver function tests (LFTs), and renal function tests (RFTs), were within normal limits: The values for test results were as followed-Hemoglobin: 13.4 g/dL (Normal: 12-15 g/dL), White Blood Cell Count: 6,200/µL (Normal: 4,000-11,000/µL), Platelet Count**:** 250,000/µL (Normal: 150,000-450,000/µL), Serum Creatinine: 0.8 mg/dL (Normal: 0.6-1.2 mg/dL**),** Liver Enzymes**:** Within normal limits, Electrolytes**:** Normal, Coagulation Profile**:** Normal

A lumbar puncture was not performed as there were no signs of meningism or raised intracranial pressure.

MRI of the brain with contrast revealed a well-defined, extra-axial, bilobed hyperintense lesion in the posterior fossa, occupying the prepontine and bilateral cerebellopontine cisterns. The lesion was T1WI: Hyperintense,T2WI: Heterogeneously hypointense, FLAIR: Hypointense, DWI/ADC: No significant diffusion restriction, Postcontrast scans: No contrast enhancement. The lesion encased the basilar artery without evidence of significant vascular compromise or brainstem compression.

Given the lesion's atypical imaging characteristics and lack of definitive diffusion restriction, differential diagnoses included white epidermoid cyst, dermoid cyst, and a proteinaceous cyst. The patient was counseled about the need for surgical excision for definitive diagnosis and symptomatic relief. However, she declined surgical intervention due to personal reasons and opted for conservative management. She was started on symptomatic treatment, including: NSAIDs (ibuprofen 400 mg as needed) and acetaminophen (650 mg as needed) for pain management, multivitamin supplements, including B-complex and folate and was advised for MRI follow-ups at every 6 months interval to monitor lesion progression. At the 6-month follow-up, the patient reported a reduction in headache intensity with conservative management. Repeat MRI showed no significant change in lesion size. She continues to be monitored for any signs of neurological deterioration that may necessitate surgical intervention.

### Imaging findings

T1WI sagittal images showing a well-defined heterogeneously hyperintense lesion in prepontine cistern causing posterior displacement and indentation of brain stem and cervicomeduallary cord. T1 axial fat sat image shows a bi-lobulated hyperintense lesion in the midline posterior fossa involving prepontine and bilateral cerebellopontine cisterns with an encasement of the basilar artery. Axial T2WI show a heterogeneously hypointense lesion in the prepontine cistern with lateral extension into the bilateral cerebellopontine angle cisterns. The mass lesion encases the basilar artery. Flair axial image shows similar bilobed hypointense lesion. DWI (with ADC map) shows no diffusion restriction.3DT1 sagittal post-contrast image shows no post-contrast enhancement.

## Discussion

Epidermoid cysts are rare, benign, slow-growing extra-axial lesions that arise from ectodermal remnants during early embryogenesis. They constitute approximately 1% of all intracranial tumors and predominantly occur in the cerebellopontine angle and suprasellar regions. While the classical epidermoid cyst follows the signal characteristics of cerebrospinal fluid (CSF) on MRI, atypical “white” epidermoid cysts present unique diagnostic challenges due to their unusual imaging characteristics. The present case underscores the importance of recognizing these atypical imaging features and highlights the diagnostic dilemma posed by such lesions.

White epidermoid cysts are distinct from classical epidermoid cysts due to their hyperintensity on T1-weighted imaging (T1WI) and hypointensity on T2-weighted imaging (T2WI). This paradoxical signal behavior is attributed to the lesion's high proteinaceous and lipid content, leading to increased viscosity. The “shading sign,” a gradient of signal loss on T2WI, has been described as a characteristic feature of white epidermoids and is thought to result from variations in the cyst's internal composition [1,2]. Unlike classical epidermoid cysts, which typically demonstrate restricted diffusion on diffusion-weighted imaging (DWI), white epidermoids lack significant diffusion restriction, making them easily confusable with dermoid cysts and other proteinaceous lesions [3].

**Fig. 1.**
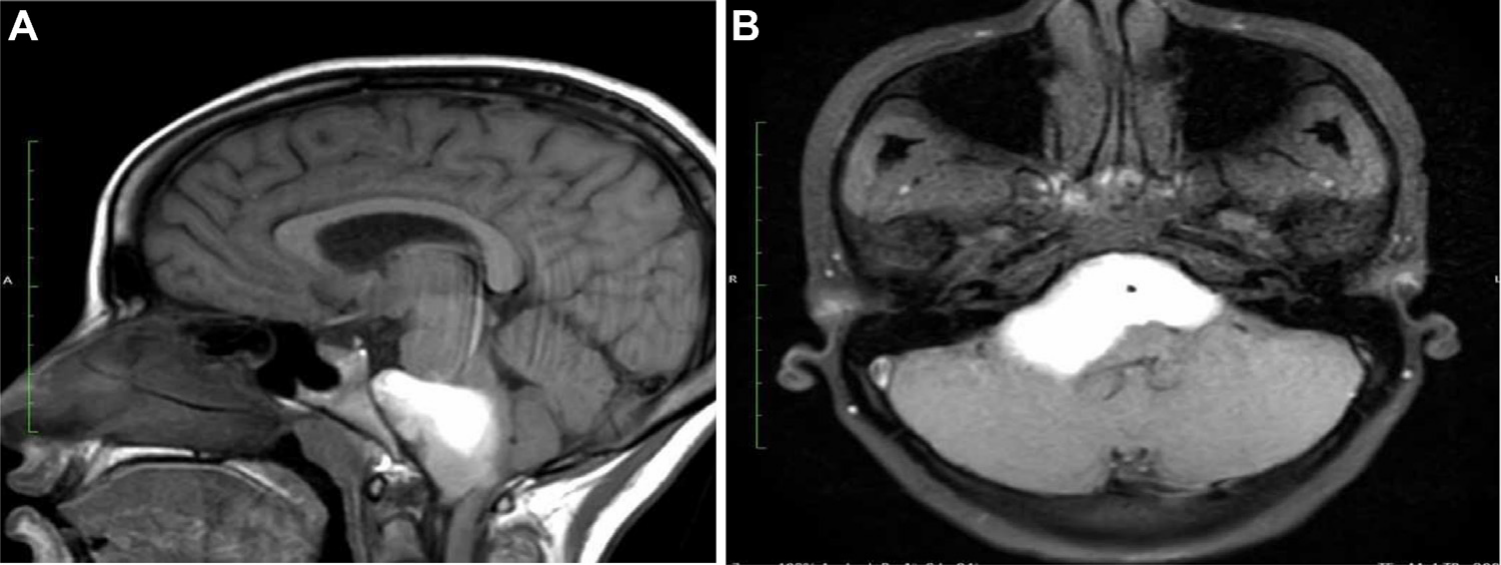
(A) T1 sagittal scan shows a heterogeneously hyperintense lesion in posterior fossa in prepontine cistern with compression and indentation of brain stem and cervicomedullary cord. (B) Axial T1WI shows a bilobed hyperintense lesion in the posterior fossa occupying the prepontine cistern and bilateral cerebellopontine cistern. The basilaral artery (black arrow) is encased.

**Fig. 2:**
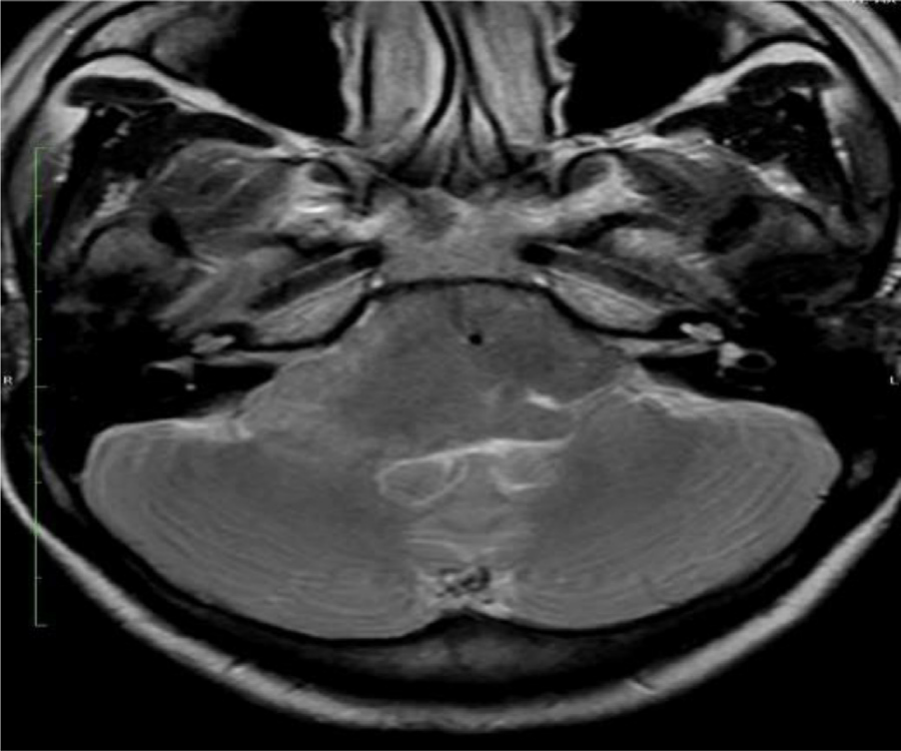
Axial T2WI show homogenously hypointense lesion in prepontine and bilateral cerebellopontine cisterns with encasement of the basilar artery.

**Fig. 3:**
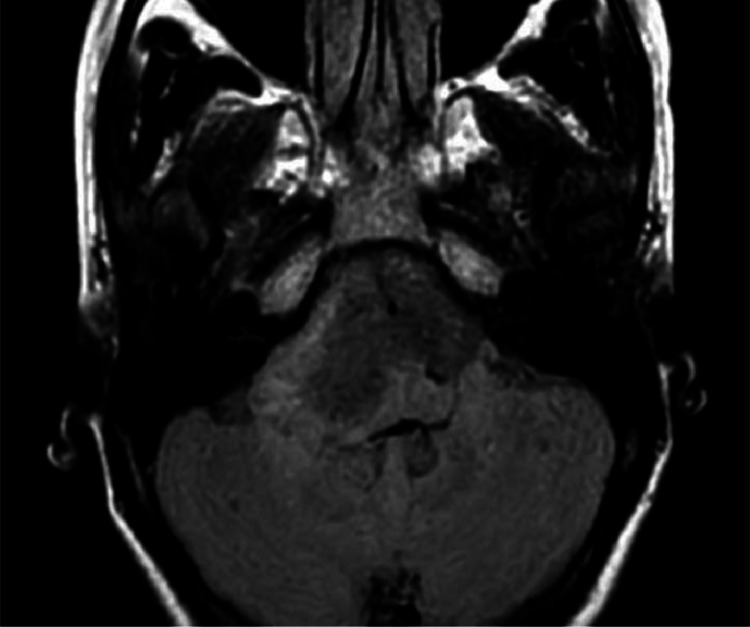
Axial FLAIR image shows a suppression of contents.

**Fig. 4:**
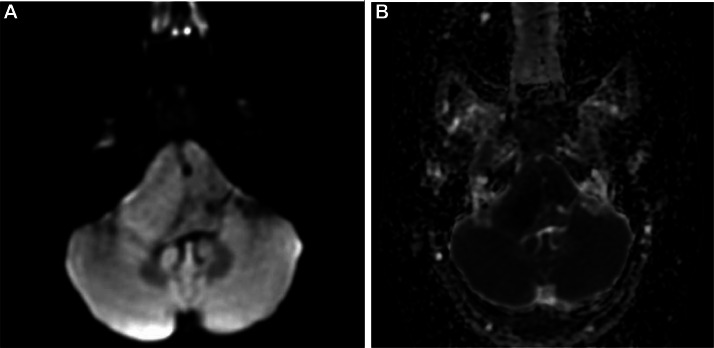
(A) DWI and (B) ADC map, show No diffusion restriction within the contents of lesion.

**Fig. 5:**
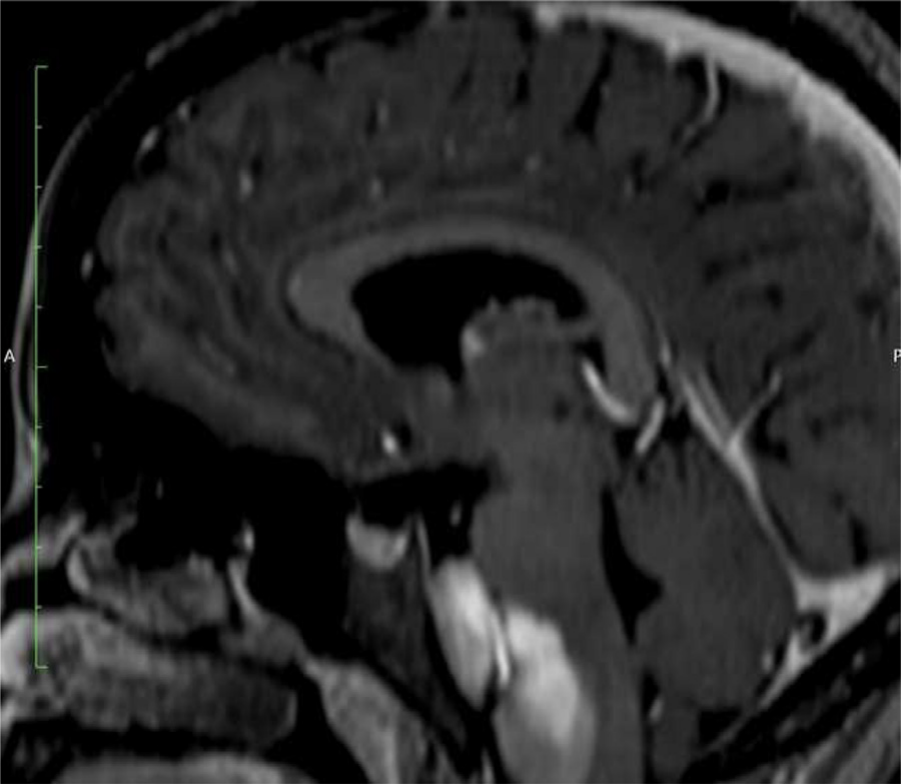
3DT1 sagittal post contrast image show no contrast enhancement.

A key differentiating factor between epidermoid and dermoid cysts is the presence of dermal appendages such as sebaceous glands and hair follicles within dermoid cysts, which are absent in epidermoid cysts. This distinction is crucial as dermoid cysts often contain fat, leading to chemical shift artifacts and fat suppression on MRI [4]. The absence of contrast enhancement further supports a diagnosis of a non-neoplastic cystic lesion. The other less likely differentials are colloid cyst and neurocysticercosis in endemic areas. Colloid cysts are typically found in the third ventricle, and can also appear hyperintense on T1-weighted imaging due to their mucinous and proteinaceous content. However, they are usually located in the anterior aspect of the third ventricle and can cause obstructive hydrocephalus, leading to episodic headaches, which was not observed in this case. In endemic regions, NCC should be considered, particularly in its colloidal-vesicular stage, where it can appear hyperintense on T1WI. Unlike epidermoid cysts, NCC often presents with peripheral edema, enhancement, and a scolex within the lesion. Additionally, serological tests and a history of exposure to Taenia solium can aid in differentiation (Fig. 1., Fig. 2, Fig. 3, Fig. 4, Fig. 5).

The differentiation of white epidermoid cysts from other cystic lesions is crucial for accurate diagnosis and management. Below is a comparative table summarizing the key imaging differences:

**Table:** MRI characteristics of different cystic lesions

Feature	White epidermoid cyst	Classical epidermoid cyst	Dermoid cyst	Colloid cyst	Neurocysticercosis (colloidal stage)
T1WI	Hyperintense	Hypointense	Hyperintense (due to fat)	Hyperintense (proteinaceous)	Hyperintense (proteinaceous)
T2WI	Hypointense	Hyperintense	Heterogeneous	Variable	Hyperintense
FLAIR	Hypointense	Hyperintense	Hyperintense	Hyperintense	Hyperintense with edema
DWI	No restriction	Restriction present	No restriction	No restriction	No restriction
Contrast enhancement	None or minimal	None	Rim or nodular enhancement	May enhance	Ring enhancement
Location	Extra-axial (cisterns)	Extra-axial (cisterns)	Midline, extra-axial	Third ventricle	Parenchymal, subarachnoid
Additional features	Shading sign, high viscosity	Follows CSF signal, scalloped margins	Fat content, possible rupture	May cause obstructive hydrocephalus	Scolex visible in some cases

Although most intracranial epidermoid cysts remain asymptomatic for years, progressive growth can lead to compressive symptoms, including headache, cranial nerve deficits, and seizures. In the present case, the patient's persistent headache likely resulted from mass effect on adjacent neurovascular structures. The involvement of the prepontine and cerebellopontine angle cisterns with encasement of the basilar artery raises concerns about potential vascular compromise and brainstem compression, which may lead to more severe neurological deficits if left untreated.

Surgical resection is the preferred treatment for symptomatic cases, particularly when there is a risk of compression of critical structures. However, complete excision can be challenging due to adherence to neurovascular structures, and subtotal resection may be preferred to minimize surgical morbidity. Incomplete removal carries a risk of recurrence, and long-term follow-up with serial imaging is essential [5,6].

One of the most serious complications associated with epidermoid cysts is rupture, which can lead to aseptic meningitis due to the release of irritant keratinous material into the subarachnoid space. This inflammatory response can result in severe headache, fever, and chemical meningitis, necessitating aggressive corticosteroid and symptomatic management. White epidermoids, in particular, are more prone to rupture compared to classical epidermoids due to their higher internal pressure and viscosity [7].

Malignant transformation of epidermoid cysts into squamous cell carcinoma, although rare, has been reported in the literature. The risk of malignancy is estimated to be between 0.011% and 0.045% and is often associated with long-standing untreated cysts [8]. Imaging features suggestive of malignant transformation include rapid cyst enlargement, heterogeneous enhancement, and infiltration into adjacent structures.

### Limitations

Despite the detailed imaging findings and clinical correlation, this case report has certain limitations. The most significant limitation is the absence of histopathological confirmation, which remains the gold standard for diagnosis. Although MRI characteristics strongly suggest an atypical white epidermoid cyst, definitive diagnosis requires tissue sampling. Additionally, the patient declined surgical intervention, limiting further assessment and potential curative treatment. Another limitation is the reliance on follow-up imaging rather than histopathological evidence to confirm stability or progression of the lesion. This raises challenges in distinguishing rare mimickers, such as xanthogranulomas or unusual proteinaceous cysts, which can have overlapping imaging features.

## Conclusion

This case highlights the diagnostic challenges posed by atypical “white” epidermoid cysts and the importance of considering them in the differential diagnosis of hyperintense T1WI lesions. The absence of diffusion restriction on DWI and hypointensity on T2WI should alert radiologists to this rare entity. Although histopathology remains the gold standard for definitive diagnosis, characteristic imaging findings can guide clinical decision-making, especially in cases where surgical intervention is deferred. However, the absence of histopathological confirmation remains a major limitation in this case, as definitive tissue diagnosis could have provided more certainty regarding the lesion's nature. Close radiological follow-up is warranted in such cases to monitor for lesion progression or complications.

## Patient consent

The patient was informed and consent for publication was obtained.
